# Multi Jet Fusion PA12 Manufacturing Parameters for Watertightness, Strength and Tolerances

**DOI:** 10.3390/ma11081472

**Published:** 2018-08-18

**Authors:** Sergio Morales-Planas, Joaquim Minguella-Canela, Jordi Lluma-Fuentes, Jose Antonio Travieso-Rodriguez, Andrés-Amador García-Granada

**Affiliations:** 1 Fluidra, Av. de Francesc Macià, 60, 08208 Sabadell, Spain; smorales@fluidra.com; 2 Department Mechanical Engineering, Universitat Politècnica de Catalunya, Av. Eduard Maristany, 16, 08019 Barcelona, Spain; joaquim.minguella@upc.edu (J.M.-C.); jordi.lluma@upc.edu (J.L.-F.); antonio.travieso@upc.edu (J.A.T.-R.); 3 GEPI-IQS Grup Enginyeria Producte Industrial, Universitat Ramon Llull, Via Augusta, 390, 08017 Barcelona, Spain

**Keywords:** porosity, Multi Jet Fusion, fluid handling, watertightness, 3D printing, anisotropy, printing orientation, water pressure, leakage

## Abstract

The aim of this paper is to explore the watertightness behaviour for high pressure applications using Multi Jet Fusion technology and polyamide 12 as a material. We report an efficient solution for manufacturing functional prototypes and final parts for water pressure applications and provide manufacturing rules for engineers in the pressurized product development process for up to 10 MPa of nominal pressure. The research findings show manufacturers the possibility of using additive manufacturing as an alternative to traditional manufacturing. Water leakage was studied using different printing orientations and wall thicknesses for a range of pressure values. An industrial ball valve was printed and validated with the ISO 9393 standard as also meeting tolerance requirements. This paper is a pioneering approach to the additive manufacturing of high-performance fluid handling components. This approach solves the problem of leakage caused by porosity in additive manufacturing technologies.

## 1. Introduction

Additive manufacturing, also known as 3D printing, converts a computer-aided three-dimensional model (CAD) to a physical object without the need for moulds or tooling. It transforms the manufacturing method from analogical to digital [1]. The manufacturing process is based on the addition of material combined with a contribution of energy to create a solid, layer by layer. Additive manufacturing technologies lead to new manufacturing paradigms such as decentralized manufacturing or mass customization. However, the transition from Rapid Prototyping (RP) to Additive Manufacturing (AM) entails new challenges in the mechanical and material fields [2]. 

The HP Multi Jet Fusion™ technology, henceforth MJF, was designed to embrace the market niche for both functional prototyping and end-part manufacturing in the industrial field. MJF is able to achieve similar results to plastic transformation processes such as plastic injection moulding. It is considered a High-Speed Sintering (HSS) technology [3], as it involves the sintering of 2D profiles of layers of powder, similar to Binder Jetting (BJ) technology, without the need for a laser. The MJF printhead prints functional agents in precise locations onto the material to define the geometry of the part and its properties. It is capable of printing thirty million drops per s across the width of the printing space [4]. This leads to very accurate dimensional precision (±0.2%) compared with other technologies [5]. The printing velocity, the dimensional precision, and the high quality in printed parts turn this novel technology into an interesting manufacturing system for AM solutions.

The aim of this study is to show how the MJF technology can be used to fabricate both functional prototypes and small batch series loaded with interior water pressure. Other additive manufacturing technologies do not achieve watertightness, due to poor mechanical properties or the porosity structure of the material. There is a need in the fluid handling industry to manufacture parts through AM technologies resistant to pressure and impermeable to leaks. As for the requirement of tightness under fluid pressure, the weak point is the porosity generated in the manufacturing process. Currently, coatings or infiltrations are required to seal the material and ensure no leakage through porosity [6].

Due to the nature of the process, technologies such as Fused Filament Fabrication (FFF) or Selective Laser Sintering (SLS) [7] create porosities placed at the interface between the layers [8]. Moreover, the manufactured parts are not completely sealed which lead to alterations in their mechanical properties [7,8,9,10,11] even as a function of time [12]. Recent studies have tried to relate laser additive processing to functionality [13]. Even though the resulting parts from SLS are not entirely dense, very low degrees of porosity can be obtained by adjusting the printing parameters [14]. This porosity is created by defects in the sintering of the material, where some of the grains do not fuse, producing empty spaces in the subsequent cooling process [15]. Two types of porosities are created: irregular pores caused by shrinkage and non-fused parts and spherical pores that come from trapped gases or evaporation of the material [16]. 

On the other hand, technologies based on photosensitive resins such as Stereolithography (SLA) and Poly Jet (PJ) do not create porosities in their structure. The resins used are formed by acrylate groups, which crosslink quickly, and epoxy groups, which enhance the mechanical properties and reduce shrinkage and curling [16]. The main problem with SLA for functional parts is not the liquid resin, but the poor mechanical properties of the crosslinked polymer. Namely, the parts are too brittle for most engineering applications. The degradation of the material caused by aging directly affects the mechanical properties [13,14]. Photosensitive resins are not recommended as a final solution, although they are very useful in the design phase [15,16,17]. In addition, these types of resins increase their volume in contact with water or moisture. For this reason, they cannot be used as the final product as they would lose all their manufacturing tolerances [18,19]. However, we should differentiate the liquid resin (monomer) and solid crosslinked polymer for each study. Recent studies focus on parameters to obtain a uniform deposition of material [20] with different additive manufacturing techniques.

Another issue is the sealing of the printed parts, which presents porosity in their structure. There are already existing solutions, where the choice of the solution depends on several factors, such as cost, machinery, and process time, among others. The selected solution has a direct impact on the time spent per part and, consequently, its cost. Therefore, when the part is analysed and requires a sealing treatment, it is replaced by the conventional plastic transformation processes due to the high industrial cost [21]. The most used sealing methods in the additive manufacturing industry are [22].

Painting and filling: When parts need only partially sealed surfaces, a few coats of paint and a little body putty can be an inexpensive option. Since this is a manual operation, the accuracy and quality of the product is influenced by the technician’s skill and care. The advantages of this option include low cost, short cycle time and ease of application. Its disadvantages are the lack of an airtight seal and inability to resist high temperatures and chemicals.

Smoothing station: This method seals the surfaces of a part by exposing them to a vaporized smoothing agent inside a chamber. The smoothing station is very easy to use and preserves dimensional integrity [23]. Its use is limited to applications with pressures that do not exceed atmospheric pressure and temperatures equal to or below 100 °C. This technique only seals the surface holes but not the internal channels. If the sealing of the interior is required, it should be combined with a previous infiltration.

Solvent dipping: Dipping additive manufacturing parts in a solvent could be a substitute for the smoothing station, when it is unavailable or the part exceeds the chamber capacity. All the characteristics are the same as the smoothing station except the dimensional accuracy, which is lower. The solvent melting action is quick and aggressive, so dimensional accuracy is difficult to control. As with the smoothing station, the use of this method should be limited to low temperature and atmospheric pressure applications.

Thermal post-treatment: Typically between the Tg and Tm of the polymer used. Thermal post-treatment can also be used with one of the other mentioned processes (coatings or infiltration) to harden the infiltrant and increase the ceiling temperature of the end part.

Adhesives coatings and infiltrations: These are substances based on epoxy formulation with different viscosities. Adhesives of high viscosity should be applied with a surface coating. In contrast, low viscosity infiltrations can be performed in vacuum chambers in order to ensure the adhesive enters to the interior of the part by controlling the necessary process time [24]. By applying adhesives to parts manufactured with SLS and FFF technologies, resistance to water pressure can only reach a value of 0.45 MPa [25].

The results reached in this work are important for the industry, because they show manufacturers the possibility of considering additive manufacturing as an alternative to traditional manufacturing methods, especially for the production of spare parts and small batches including mass customization for pressurized components. This paper is a novel approach to designing and manufacturing fluid handling components through additive manufacturing Multi Jet Fusion technology while avoiding the problems of water leakage. This problem is common in most of the additive manufacturing technologies and is caused by porosity structures in the material.

## 2. Materials and Methods 

The fabrication of a valve through MJF technology with PA12 is presented in this study. PA12 powder was provided by 3D HP Jet Fusion 4200 (Hewlett Packard, Barcelona, Spain) with the following specifications (Table 1):

Specifically, a ball valve designed to isolate a liquid belonging to a conducting fluid system following the standard EN ISO 16135:2007 [26] and the directive 97/23/EC [27]. The valve is catalogued as PN10 (1 MPa). Nominal pressure (PN) is used as a reference for its mechanical resistance and corresponds to the maximum allowed water pressure at 20 °C.

**Figure 1:**
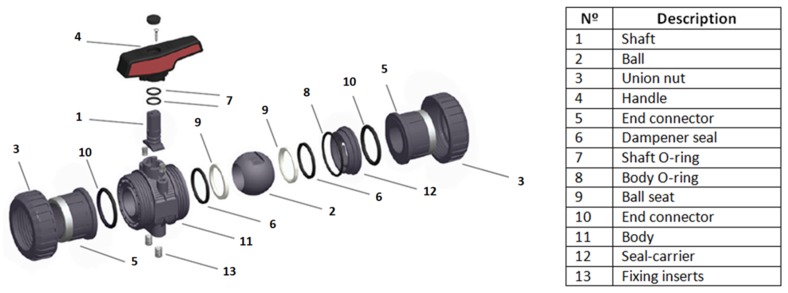
Exploded view of ball valve.

For the sake of simplicity, only the main parts of the valve (shell and union nuts) were considered to be printed with MJF (11 and 3, respectively, in Figure 1). These exterior coverage parts support two pressure origins: the pressure from the inner parts and the hygroscopic pressure.

The printed parts were evaluated with the same quality tests applied to an industrial production valve. It must satisfy ISO 9393-1 [28]. This standard describes the method to verify the shell resistance under water pressure and the inner parts package effort. Together with the union nuts, they must withstand the tensile strain caused by the water pressure inside the valve and comply with ISO 228-1 [29].

The methodology used was divided into three stages. In the first stage, referred to as the fabrication process, the three-dimensional model was analysed. It was treated in the printing software. The thickness of the layer, the material used and the orientation of the parts in the space were set up. Before the printing process, the material was subjected to a tensile test to correctly characterize its mechanical properties. Also, a fractography analysis was done to characterize the porous media. Furthermore, a leaks study was implemented by using a flowmeter, given different printing orientations and wall thickness for a range of pressure values. In the s stage, the physical dimensions of the parts were verified by using a three-dimensional scan. This compares their dimensions with the tolerances allowed in the production of the valves. In cases where tolerances are not satisfied, machining of the parts must be performed. The full valve assembly will be checked with a leak test using air as a fluid. The final stage is product validation. This determines whether the complete valve satisfies the quality standard for valves made of thermoplastic material.

A 3D HP Jet Fusion 4200 (Hewlett Packard, Barcelona, Spain) was used to manufacture the parts of the valve. The printing parameters used were: 0.08 mm of layer height in the Z axis with a standard resolution of ±0.2%. A balanced print mode was set up: One rolling step and two injection passes spending 10.5 s per layer. Due to the shape of the parts, the cylindrical sections were oriented in the XY plain, where the resolution is highest. In the XY plane orientation, the step effect caused by an angular gradient lower than 30° in the Z axis is avoided.

Once manufactured, the parts were dimensionally analysed using a 3D scan (ATOS Scanbox 4105, Leuven, Belgium). Then the set was assembled in order to validate the product. The next stage was identifying whether there were any leakage points throughout the leak tester.

In this case, the shell test and the seat and packing test were studied. These tests provide information about the resistance and watertightness of the material. To carry out the tests, there are some necessary restrictions, as shown below:

- Pressure appliances, as specified in ISO 1167-1, have to be able to connect the sample and progressively apply water pressure following the standard of the product. It has to maintain a constant pressure between +2% and −1% for the time specified in ISO 9393-2 [30], maintaining the temperature indicated in the product standard.
- Pressure calibrated sensors must be able to verify the test specified pressure without polluting the product.
- Thermometers must be able to verify the specified temperature in the assay.
- Timers have to be able to record the duration of the pressure application until the fail momentum during the trial time.

The procedure established for the shell test is as follows:

1. In the first place, the sample must be filled with water and conditioned for at least 1 h at a temperature that does not deviate by more than ±2 °C from the specified trial temperature.
2. Place the test sample in a mode where the entire valve body is under the trial pressure.
3. Make sure that the water temperature in the test tube is adjusted to the specific trial temperature.
4. Release any trapped air inside the trial sample.
5. Raise the pressure progressively until the trial pressure specified in ISO 9393-2 [30] is reached; this should be done as fast as possible, but not in less than 30 s.
6. Maintain the pressure and temperature for the duration specified in the standard ISO 9393-2 [30].
7. Diminish the pressure until atmospheric pressure is reached.

The ISO 9393-2 [30] parameters of the trial according to the fabrication thermoplastic material are specified in detail. Since PA 12 is not included in the standard, the test parameters for the PVC-U, which is the original material of nut unions and shells, as well as being the most restrictive case, were used. The shell test parameters are shown in Table 2.

The procedure established for the seat and packing test is specified below:

Firstly, the sample must be filled with water and conditioned for at least 1 h at a specified temperature, which does not deviate more than ±2 °C.

1. Fully closed valve test:
  - Connect one end of the sample to the pressure line and the other end to a device capable of detecting leakage.
  - Fill the closed sample with the test fluid at the specified temperature.
  - Release any trapped air from the test sample.
  - Close the valve with the closing torque specified in the product standard.
  - Increase the pressure gradually until reaching the test pressure specified in ISO 9393-2 [30], but not in less than 30 s.
  - Maintain the pressure and temperature duration specified in ISO 9393-2 [30].
  - Check the seat tightness.
  - Reduce the pressure to atmospheric pressure.
2. Valve test totally or partially open:
  - Open the valve to such an extent that all related cavities and packings are under the test pressure.
  - Connect one end to the pressure supply and close the other end.
  - Fill the sample with the test fluid to the specified temperature and then close the flow downstream of the test sample.
  - Release any trapped air from the test sample.
  - Increase the pressure gradually until reaching the test pressure specified in ISO 9393-2 [30], but not in less than 30 s.
  - Maintain the pressure and temperature duration specified in ISO 9393-2 [30].
  - Check the body and packing tightness.
  - Reduce the pressure to atmospheric pressure.

The procedure determined for the seat and packing test and the corresponding parameters are specified in detail in the ISO 9393-2 [30], and they are shown in Table 3.

## 3. Results

### 3.1. Tensile Tests

**Figure 2:**
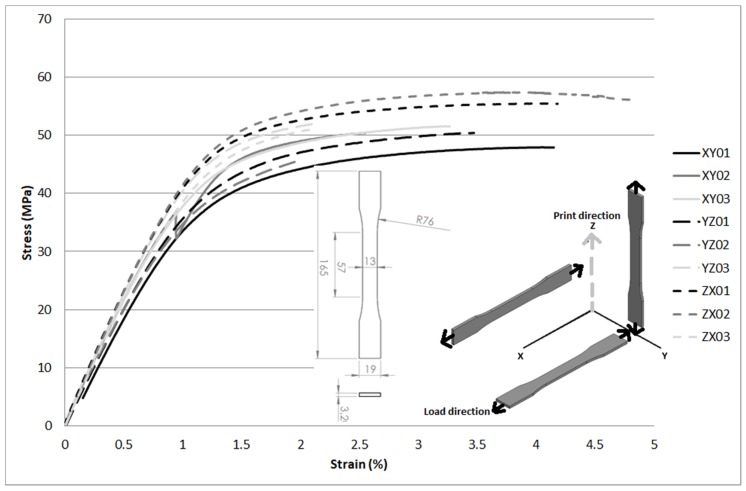
Tensile tests for several fabrication directions using D638 Type I specimens.

Tensile tests were carried out on specimens according to standard ASTM D638 [31] Type I. They were manufactured on three different printing directions, and stress versus strain curves were obtained. For each orientation, three specimens were tested. The results from such tensile tests are shown in Figure 2.

The mechanical properties were obtained from the tensile test curves. Furthermore, a comparison between these measurements and the reference values of polyamide resins and vinyl compounds are provided in Table 4. For each specimen, Young modulus (E), yield stress (σ_y_), maximum stress (σ_m_) and rupture strain (ε_r_) are reported.

According to Galileo-Leibnitz, also known as the Clebsch-Rankine criteria [34], the maximum stress is calculated. The maximum stress for polyamides, according to Russian GOST normative 10589–63 [32], is within the range of 49–59 MPa approximately. The experimental results obtained were in the range 48–57 MPa. There is not a clear relation between fabrication direction and maximum stress. The observed rupture for all fabrication directions was cohesive and fragile, with ultimate strain lower than 5% in all cases. 

In addition, an Analysis of Variance (ANOVA) test was performed, taking into account the maximum stress for the three populations: XY, YZ and ZX. The obtained results showed that there is no strong evidence to reject the null hypothesis, which considers that all the specimens belong to the same population, as the obtained p-value was 0.135. However, this result was partly expected, due to the low number of specimens for each population (3 specimens for each orientation). Specimens were directly printed for each direction, and did not require further manufacturing. For other techniques, the manufacturing of coupons might affect the results [35]. 

### 3.2. Leaking and Pressure Tests

Leak tests were carried out with vessel samples made of PA12 with horizontal (H) and vertical (V) printing orientations. The inner diameter was a constant parameter (25 mm) for all the samples, with the wall thickness being the parameter to be modified in the range from 0.4 mm to 0.7 mm. The wall thickness was only modified in the cylindrical zone with a length of 50 mm. All tests were performed using a flowmeter to measure leaks at different pressures of up to 0.4 MPa. 

**Figure 3:**
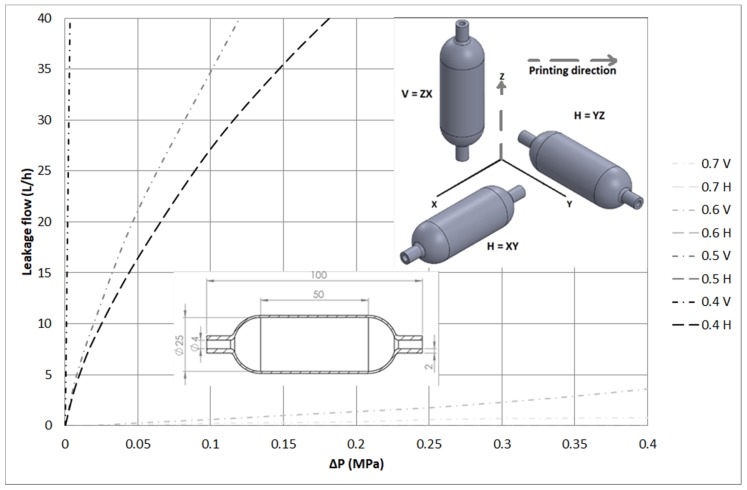
Leakage flow rate and pressure drop for all specimens.

Test results show that, for samples printed with the same orientation, lower thickness leads to higher leak values. On the other hand, for samples with the same wall thickness, the orientation has a great impact on the leak values, with the vertical orientation being the most critical. This is related to the number of layers and the air gaps that can form during the printing process. Concerning the printing height, the number of layers is greater with the vertical orientation; therefore, the probability of air gaps forming during the printing process is higher. Figure 3 shows the design of the samples, as well as the leak values at different pressures. 

For a leak value of 3 L/h, the pressure drop (ΔP) values have been calculated in Table 5. It shows that both printing orientation and thickness are significant variables that affect leakage values.

**Figure 4:**
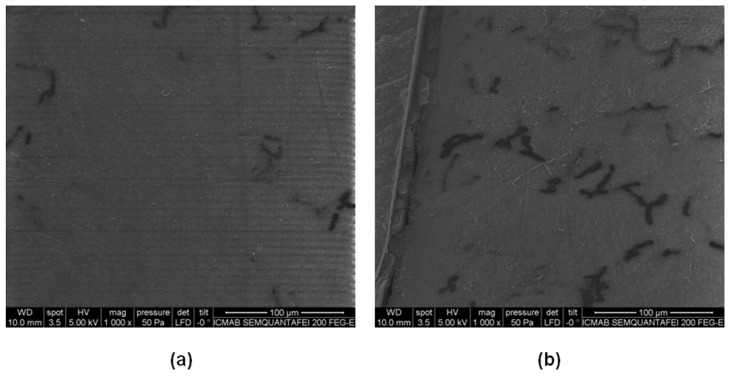
(**a**) SEM in XY orientation, (**b**) and YZ orientation.

This information is relevant for design engineers during the design phase. The design rules presented are very useful for making decisions about the dimension and printing strategy of the pressurized component. In Figure 4, two samples with different orientations, but with the same wall thickness, are sectioned and visually inspected through a fractography analysis. The quantity of pores found in the vertical orientation is much higher. It was concluded that the printing orientation plays a significant role in the creation of pores and consequently in the leakage.

### 3.3. Tolerances on Final Parts

The geometrical tolerances of all fabricated parts were checked and the distribution curves were obtained. The expected results for the standard resolution of the machine were satisfactory. Both parts were adequate in terms of tolerances, with dimensions varying ±0.3 mm. For this dimension range, machining of the parts was not necessary.

### 3.4. Shell Test on Final Assembly

A pressure study was required prior to shell testing on the final assembly. Depending on the printing orientation, the efforts required for the part are different. In XY and YZ orientation, or horizontal direction, the stress applied during the trial is shown in Equation (1).

(1) $$\sigma \cdot 2\cdot t=P\cdot 2\cdot r\to \sigma_{XY/YZ}=\frac{P\cdot r}{t}$$

where, *σ* = stress (MPa), *t* = thickness (mm), *r* = radius (mm), *P* = pressure (MPa).

In ZX orientation, or vertical direction, the stress applied during the trial is shown in Equation (2).

(2) $$\sigma \cdot 2\cdot \pi \cdot r\cdot t=P\cdot \pi \cdot r^{2}\to \sigma_{ZX}=\frac{P\cdot r}{2\cdot t}$$

In this case, the cylindrical section was oriented in the XY plane, where the resolution is higher. In this trial, the pressure increased exponentially until it reached the set point (4.2 MPa). Following Equation (2), the total stress applied was 15.8 MPa. The security factor for PA12 is about 2.5. Therefore, there is no risk of explosion.

**Figure 5:**
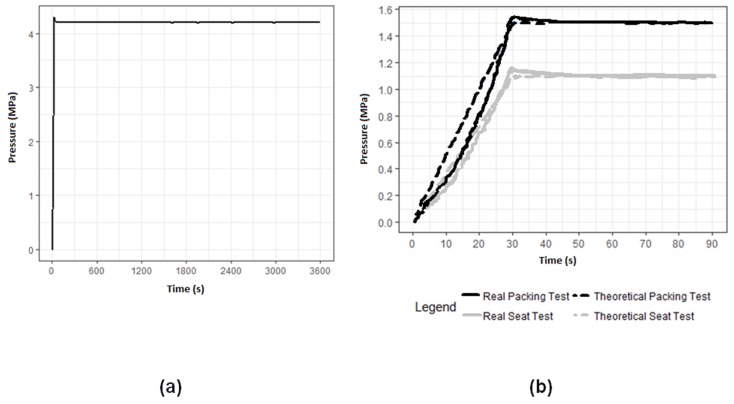
(**a**) Shell test results; (**b**) Seat packing test results.

The pressure control system acts on the pump and the pressure multiplier to reduce the pressure until the set point. A stabilization time of thirty seconds was programmed. During this time, the pressure increases, and the environment is stabilized to the set point. The one-h test starts when pressure and temperature are constant. In this process, a non-significant pressure decrease is observed due to material expansion. It is placed in the elastic section, creating empty gaps and consequently diminishing the system pressure. Pressure deviations are not detected during the test process. The results were satisfactory, as no type of leak or valve explosion was found in the sample. Figure 5 shows the time series of the pressure evolution. As to the seat and packing assembly, the results were satisfactory in that no leakage was observed through the valve seat and packing during the trial time, as shown in Figure 5. In both cases, there was noise in the pressure measurement during the trial test time due to the sensor. The variation was approximately ±3 kPa.

## 4. Conclusions

Considering the performed tests and the analysis of the results, several conclusions can be drawn:

1. The MJF technology can encompass the market niche for both the functional prototype and the end parts in the fluid handling field. This technology is able to substitute conventional plastic transformation methods, such as plastic injection moulding.
2. This solution avoids sealing of the printed parts as a post-process for achieving the desired functionality in the fluid conduction industry. The coating and infiltration seals only allow maximum pressures of 0.45 MPa, while with MJF technology, 4.2 MPa of water pressure was achieved for one hour, due to the lack of porosity in its structure.
3. Wall thickness and printing orientation are key variables which determine the watertightness of the samples. For the same wall thickness, vertical orientation requires higher number of layers to be printed, and therefore is more likely to have air gaps that lead to greater leak values. Test results show that for samples printed with the same orientation, a lower thickness leads to higher leak values.
4. Watertightness was validated through a real case: An industrial ball valve. The tests under the standard pressures of the shell and packaging/seat were satisfactory, showing no leaks and therefore comply with the quality control specified for PN10 ball valves made with thermoplastic material.

## Figures and Tables

**Table 1:** Powder specifications.

Property	Value	Normative
Powder melting point (DSC)	187 °C	ASTM D3418
Particle size	60 µm	ASTM D3451
Bulk density of powder	0.425 g/cm^3^	ASTM D1895
Density of parts	1.01 g/cm^3^	ASTM D792.

**Table 2:** Shell test parameters for PVC-U material.

Material	Minimum Test Time	Pressure Test (P_test_)^1^	Design Stress (σ_t_)^2^	Assembly Stress (σ_s_)^3^	Temperature	Inner Fluid	Outer Fluid
PVC-U	1 h	4.2 MPa	42 MPa	10 MPa	20 ± 2 °C	Water	Water or Air

**^1^** The test pressure is measured following the formula: P_test_ = (σ_t_/σ_s_); ^2^ Design stress corresponds to the maximum stress for the elastic limit not to be exceeded avoiding its plastic strain (MPa); ^3^ Stress induced under the assembly conditions (MPa).

**Table 3:** Seat and packing test parameters.

Test	Test Minimum Time	Test Pressure (P_test_)	Temperature	Inner Fluid	Outer Fluid
Seat test (close valve)	DN ≤ 200 = 15 s	1.1 MPa	20 ± 2 °C	Water	Air
Packing test (open valve)	DN > 50 = 30 s	1.5 MPa	20 ± 2 °C	Water	Air

**Table 4:** Values obtained from tensile tests and reference values from Russian GOST normative 10589-63 [32] for PA and 9639-61 [33] for PVC-U.

Specimen	E (GPa)	σ_y_ (MPa)	σ_m_ (MPa)	ε_r_ (%)
XY01	3.525	33.5	47.9	4.1
XY02	4.202	35.2	50.3	2.5
XY03	4.087	37.7	51.6	3.3
YZ01	3.817	35.5	50.3	2.5
YZ02	3.767	34.6	45.6	2.0
YZ03	4.321	40.4	52.1	2.2
ZX01	4.391	41.5	55.4	4.2
ZX02	4.409	41.4	57.4	4.8
ZX03	4.106	40.1	50.9	2.1
PA (GOST 10589-63) [32]	1.167	-	49.0–58.8	100
PVC-U (GOST 9639–61) [33]	2.942–3.923	–	39.2–58.8	10–100

**Table 5:** Pressure drop for a leak value of 3 L/h.

Thickness (mm)	Printing Orientation (H = Horizontal and V = Hertical)	Pressure to Leak 3L/h (MPa)
0.7	V	>0.400
0.7	H	>0.400
0.6	V	0.357
0.6	H	>0.400
0.5	V	0.044
0.5	H	>0.400
0.4	V	0.003
0.4	H	0.052
